# Variation in emergency department visits among residents of Swedish nursing homes between 2019 and 2020, a population-based cohort study

**DOI:** 10.1186/s12913-025-13443-9

**Published:** 2025-09-10

**Authors:** Wilhelm Linder, Douglas Spangler, Megan Doheny, Alfons Grönström, David Isaksson, Ulrika Winblad

**Affiliations:** 1 Health Services Research, Department of Public Health and Caring Sciences, Uppsala University, Uppsala, Sweden; 2 Aging Research Center, Karolinska Institute, Stockholm, Sweden

**Keywords:** Nursing homes, Long-term care facilities, Emergency departments, COVID-19

## Abstract

**Background:**

Nursing homes have limited capacity to provide medical care to clinically frail residents and therefore rely on transferring residents to hospital-based emergency departments when acute medical needs arise. The utilization of emergency department care varies between nursing homes but the extent of this variation is unexplored. Further, the effect of organizational characteristics of nursing homes on emergency department utilization is unknown. This study aims to characterize the variation in emergency department visit rates between nursing homes, and to identify contextual and organizational characteristics that contribute to this variation.

**Study design:**

Population-based cohort study of individuals living in nursing homes during 2019 and 2020 in Sweden.

**Methods:**

National registry data on nursing home residents were linked to nursing homes based on civil- and business registration addresses. Emergency department visits were identified for each resident in the national patient registry and measured as incidence rates per nursing home. Multi-level analysis was performed to investigate the association between emergency department visit rates, and contextual and organizational characteristics of nursing homes.

**Results:**

The median incidence rate of emergency department visits from nursing homes was 5.2 per 100 person-months in 2019 (IQR = 3.7–6.9) and 4.4 per 100 person-months in 2020 (IQR = 3.0–5.7). Individuals living in nursing homes in the most rural locations had lower odds of emergency department visits (OR 0.51, 95% CI 0.41–0.61 versus the most urban locations). Moreover, individuals in nursing homes specialized in dementia care had lower odds of emergency department visits (OR 0.90, 95% CI 0.87–0.94 versus somatic care).

**Conclusion:**

The results suggest that the location and organizational characteristics of nursing homes may have an impact on the utilization of medical services by the nursing home resident population. Further research is warranted to investigate any ensuing health inequities.

**Clinical trial number:**

Not applicable.

**Supplementary Information:**

The online version contains supplementary material available at 10.1186/s12913-025-13443-9.

## Background

The share of older adults is increasing in many parts of the world, placing strain on health care services. In particular, the growing population of clinically frail older adults with complex health care needs is increasing the demand for emergency department (ED) care. However, EDs are arguably unsuitable places of care for older adults, and ED visits are associated with hospital-acquired infections as well as risks related to unmet nursing care needs (e.g. falls, dehydration and delirium) [1–3]. As a result, many health care systems are intensifying their efforts to provide health care services in outpatient settings. Furthermore, since a large proportion of older patients with complex needs reside in nursing homes (NHs), this care setting is becoming increasingly important for addressing the medical needs of the ageing population. The way NHs are configured - both in terms of staffing, resources, and medical competencies - can significantly influence whether residents receive adequate care on-site or are transferred to EDs. From a health care system perspective, it is therefore urgent to improve our understanding of how organizational aspects of NHs affect health care utilization.

Although there is no consensus regarding the optimal level of ED rates for NH-residents, both high and low ED (and hospitalization) rates may indicate low quality of care [4]. A high degree of ED visits could suggest that on-site medical capabilities are underdeveloped (or underutilized), exposing NH residents to unnecessary medical risks and suffering related to potentially avoidable hospitalizations. On the other hand, low hospitalization rates may indicate unnecessary suffering from treatable conditions and possibly increased mortality [5]. Furthermore, variation in hospitalization rates among NHs within the same geographical area may contribute to health inequities due to unequal access to medical treatment.

The decision to transfer NH residents to EDs depends on several factors, with staff balancing the available medical care in the NH, the potential medical benefits and risks associated with ED visits and the wishes of the patient and their relatives [6]. Previous studies indicate that the most common reasons for seeking care in EDs among NH residents are cardiovascular disease, infections and fractures [7, 8]. Of particular concern are potentially avoidable ED visits, i.e. ED visits that could have been prevented given adequate medical services provided in a timely manner [9]. As such, several health care systems are looking at improving medical care in NHs and increasing the utilization of mobile care teams [10, 11].

Despite this, ED visits and hospitalizations for ambulatory care sensitive conditions are common among NH residents, with a large proportion of these hospitalizations considered potentially avoidable [12, 13]. One reason for this is that NHs may be limited in their capacity to provide medical care, resulting in diseases amenable to ambulatory care requiring hospital treatment. Additionally, NH residents are often clinically frail and near the end-of-life, where the benefits of being transferred to hospital may not outweigh the risks and stress associated with hospitalization. Finally, studies have shown that up to 20% of NH residents die in hospitals, prompting discussions about how to handle terminally ill patients, as surveys report a strong preference for dying at home [14, 15].

### Characteristics of nursing homes and their impact on emergency department visits

There is substantial variation in ED visits and hospital admissions from NHs, both within and between countries [16]. For instance, a Norwegian study reported that the mean annual rate of hospital admissions from nursing homes were 0.58 per nursing home bed, with a range from 0.16 to 1.49 per year [17]. In comparison, a study from Sweden reported mean annual rates of ED transfers in the range of 0 to 1.03 per bed and year [7].

Previous research indicates that the observed variation in ED and hospitalization rates mainly depends on the age, morbidity and socioeconomic status of NH-residents [18, 19]. In particular, NH residents with dementia have a lower probability of being treated in EDs [20–22]. Part of the observed variation is also attributable to differences in the risk-benefit assessment performed by physicians and registered nurses in NHs [23]. This is supported by a study showing that historical ED transfer rates were strongly associated with having a high ED transfer rate [24]. Furthermore, some reports indicate that the variation in ED visits for NH residents is attributable to local outbreaks of infections [25].

However, previous studies have also shown that organizational characteristics of NHs are associated with ED and hospitalization rates of NH residents [16, 17, 26–28]. First, large NHs have been shown to be associated with a greater need for hospital care, partly due to an increased risk of infectious disease outbreaks in larger NHs [24, 29]. Second, the medical capacity of NHs may influence the rate of hospitalization, with previous studies indicating that the level of continuity of care [30], the ability to perform advanced medical treatments [30, 31], and the ratio of medical/non-medical staff [32] can affect ED-utilization. Primary care has also been shown to be of importance as ED rates among NH-residents are lower when there is high accessibility to primary care, out-of-hours care and home visits in an area [33]. Furthermore, there is evidence that NHs with more nurse hours per resident and NHs employing care coordinators have lower ED visit rates [34]. Third, some studies have investigated management of NHs, such as differences between privately and publicly owned NHs [17, 32]. Privately owned NHs tend to have higher ED transfer rates than publicly owned NHs [34, 35], and for-profit (and private equity) NHs may have higher rates of ED transfers compared to private non-profit NHs [36, 37].

The proximity (distance) of NHs to hospitals has also been shown to influence ED utilization [24, 33]. A related factor is the geographical location of the nursing home, as outbreaks of infectious diseases, and ensuing ED visits may depend on the population density in the surrounding area. Finally, local regulations may also have an impact on ED visits and hospitalizations. For instance, restrictions in the possibility to quarantine NH residents during infectious disease outbreaks could potentially lead to an increased need for hospital services [22, 38].

### The Swedish setting

The Swedish health- and social care system offers universal access to all residents. Health- and social care is decentralized with 21 self-governing local authorities responsible for health care and 291 municipalities responsible for eldercare (including NHs for older adults) [39]. In the 1990’s eldercare in Sweden was de-institutionalized and shifted towards home care services with NHs currently being reserved for the oldest and most clinically frail individuals [40]. In 2019, there were approximately 2300 NHs (20% privately owned) for older adults with between 80 and 85 000 individuals living in NHs in a given month [41]. A majority of the NHs were specialized in dementia care, with approximately 40% specialized in care of older adults with somatic conditions [42].

NHs in Sweden share several core features with NHs in other countries, for instance in assisting with activities of daily living. However, differences in organization and on-site clinical resources may modify the rate, and patterns, of ED-transfers of Swedish NH-residents. Medical care at NHs in Sweden is mainly provided by registered nurses, nursing assistants and licensed practical nurses. In comparison to similar countries, both the level of education of staff as well as the number of registered nurses is low [43, 44]. Generally, NHs are staffed with registered nurses on weekdays (office-hours) with nurses on call during evenings and weekdays. Nurses collaborate with primary care physicians who are employed by the regional health care systems in an off-site capacity. Primary care physicians perform clinical rounds at NHs on a (bi-)weekly basis, with contacts outside of the clinical rounds mainly consisting of telephone calls between nurses and primary-care physicians on call. The collaboration between the municipal NHs and regional primary care physicians has been criticized for being inadequate, often failing to provide sufficient primary care services to NH residents [45].

Consequently, NHs in Sweden typically lack the capacity to deliver advanced medical treatments, such as oxygen therapy or drug treatments requiring infusions, and therefore depend on hospitals for managing more serious medical conditions [44]. The limited medical capacity in NHs became especially evident during the COVID-19 pandemic, when many NH-residents with severe symptoms did not receive hospital care. Instead, they remained in the NHs, where appropriate medical treatment could not be provided. In some regions, hospitals explicitly prioritized less frail patient groups, leading to under-utilization of hospital services and subsequent unmet medical needs among NH residents [46]. Taken together, the proportion of potentially avoidable hospitalizations from Swedish NHs might not be comparable to findings from other countries.

### Study rationale

NHs in Sweden have relatively limited resources to manage medical emergencies and often rely on hospitals for medical treatments of their residents. This includes treatments that could potentially be performed by primary care or other outpatient services if NHs were better integrated into the health care system. Previous research on ED visits in NH residents has mainly focused on medical and socioeconomic risk factors related to individuals. However, there is still limited knowledge regarding the impact of organizational characteristics of NHs on ED visits. Expanding this knowledge is crucial for decision-makers who are working to adapt healthcare systems to meet the needs of ageing populations and the challenges posed by future pandemics.

Therefore, this study aims to characterize the variation in ED visits from nursing homes in Sweden. The primary research objectives are:

1. To determine the extent of variation in ED visits between nursing homes.
2. To explore whether demographic and organizational differences at the municipal and NH level contribute to this variation.

The secondary research objective is to explore the effects of the COVID-19 pandemic on ED visits in relation to organizational differences between NHs.

## Method

Population-based cohort study using register data from January 2019 to December 2020 with NHs as the analytical unit.

### Study sample

The cohort of individuals used to describe NHs was derived from the Swedish Social Service Register, maintained by the National Board of Health and Welfare (NBHW). The registry contains monthly observations on all individuals in Sweden receiving municipal services such as home care and living in NHs [47]. Street-addresses of NH residents were available for all individuals who were living in NHs in December of 2018, 2019 or 2020. The study population was therefore a dynamic cohort, with individuals entering the study cohort starting in December 2018 (January 2019) up until December 2020. NH-residents were followed until December 2020 or until the month of death if that occurred before December 2020. Registry data about NH residence, ED visits and mortality was collected at the end of each month.

The following inclusion criteria were used to construct the final population of NH residents:

1. Individuals registered in the Social Service Registers as living in a NH for older adults during at least one month between in 2019-01-01 and 2020-12-31.
2. Individual having at least one known street address at the end of 2018, 2019 or 2020.
  Individual NH residents were then matched to NHs that had:
3. At least one street-address in the Business Register (Statistics Sweden) or the Unit Survey (NBHW).
4. Reported data to the Unit survey (NBHW) conducted in 2019.

NHs represented by few individuals in relation to NH-size were excluded due to concerns that the number of observations were not sufficient to represent entire NHs. This corresponded to NHs where the ratio of NH-residents / NH-size was less than 0.5 (*n* = 50).

### Outcome

ED visits for NH residents during the period 2019-01-01 to 2020-12-31 were obtained from the section of the *Swedish National Patient Registry* containing specialized outpatient care.

### Nursing home organizational variables

The independent variables were selected based on theoretical assumptions about their potential impact on ED visits and obtained from The Unit Survey conducted in 2019. The Unit Survey is a recurring survey in which NHs report indicators of NH quality to the NBHW [42, 48]. The survey contains self-reported data regarding the characteristics of individual NHs (e.g. the number of beds in an NH, staff density and private / public ownership). In 2019, 2265 NHs were contacted and 2067 of NHs responded to the survey. The data reported in 2019 was used for the entire study period as the survey was cancelled in 2020 due to the COVID-19 pandemic. Missing data in the Unit Survey was imputed using single imputation (R package Mice).

#### Public / Private ownership

Ownership was categorized as either private or public. 

#### NH profile

NHs were categorized as specialized in either somatic or dementia care, indicating both the type of NH residents as well as the type of training staff receive at the NHs.

#### Size

NH size was measured as the number of NH-beds and categorized into tertiles as small, medium and large NHs.

#### Urbanicity

Urbanicity was used as a proxy variable for distance to hospital and spread of COVID-19 as population density was associated with COVID-19 infection rates [49, 50]. The indicator was defined as the degree of urbanicity of the municipality where the NH was located in. Urbanicity was calculated based on a categorization of population density in municipalities from the Swedish Agency for Economic and Regional Growth. The degree of urbanicity ranges from densely populated metropolitan municipalities (= 1) to sparsely populated rural municipalities (= 6) [51].

#### Number of nurses

Staff density was described as the number of nurses per NH-bed for weekdays and weekends. Number of nurses were then categorized into tertiles as low, medium and high number of nurses.

### Nursing home resident variables

Variables describing age, sex, and socioeconomic factors were obtained from the Swedish Population Register (RTB, Statistics Sweden) and the Longitudinal Database for Social Insurance and Labor Market Studies (LISA, Statistics Sweden) [52, 53]. Age was measured as the time from year of birth to NH admission and sex was categorized as male or female. Highest level of education obtained was categorized as primary, secondary or tertiary education, country of birth was categorized as in-, or outside of Sweden and civil status was categorized as married (or registered partner) and unmarried (including divorced and widow/widower). Morbidity was measured using ICD-codes from inpatient and specialized outpatient visits in the Swedish National Patient Register [54]. Data on drug prescriptions from the Swedish Prescribed Drug Register was used as a proxy of conditions diagnosed in primary care which are not captured in the Swedish National Patient Register (e.g. ATC-code N06D for dementia) [55, 56]. Data on individual NH residents were linked using encrypted personal identification numbers [57]. For a summary of the registers used, see supplementary table S1.

### Statistical analysis

The characteristics of NHs and their residents were described using descriptive statistics. ED visit rates were calculated as the number of ED visits per NH and 100 person months (yearly incidence rates) and as the prevalence of ED visits per nursing home and month (standardized to ED visits per 100 NH residents).

Data from individual NH residents were aggregated per NH and month, with the dataset used in the statistical analysis consisting of 24 monthly observations (January 2019 to December 2020) for each NH. ED visits from NHs were specified as the number of NH residents with at least one ED visit for that month in relation to the number of NH residents with zero ED visits. Socioeconomic variables were aggregated per month as the proportion born in Sweden, proportion of married and proportion with tertiary education.

A risk adjustment score for the likelihood of ED visit was estimated for each resident using a regularized logistic regression model implemented using the glmnet package in R [58]. For each resident, data from the 5 years preceding NH admission was collected from the NBHW inpatient and outpatient care registries, as well at the drug prescription registry together with age and sex from the Swedish Total Population register. Data was coded as the number of primary and secondary diagnosis codes, and medication prescription events, including a squared term to account for potential non-linearity. The mean risk score per NH and month was then included as a covariate in the analytical regression model.

The data had a multi-level structure with months as level 1 (to account for seasonality) and NHs as level 2. Regression coefficients were estimated using generalized mixed effect models (binomial distribution with maximum likelihood estimation). Coefficients were expressed as odds ratios (OR) with bootstrapped 95% confidence intervals (CI). Goodness of fit was assessed by pseudo r-squared and plots of predicted and observed values, see supplementary figures S1 and S2.

Model 0 included random intercept terms for NHs and month which assessed the between group variance at the NH level. Mixed models were then used to calculate crude ORs for each independent variable. The first model (M1) included M0 and NH characteristics (ownership, care profile, size and urbanicity). Model 2 was based on M1 with the addition of nurse staffing variables as potential mediators of the effects in M1. Model 3 was based on M2 and included aggregated measures of ED visit risk adjustment score (age, sex and morbidity) as well as socioeconomic covariates (country of birth, civil status and highest education obtained) for each NH.

In the final model (M4), an interaction term was added in order to account for the COVID-19 pandemic where the monthly observations during January 2019 to February 2020 were defined as pre COVID-19 pandemic and March 2020 to December 2020 was defined as active COVID-19 pandemic.

An example of the model specification in R can be seen in the supplementary material. For the NH random intercept term, variance and proportional change in variance (PCV) were estimated. The variance partition coefficient (VPC) was calculated using the latent variable method [59]. Bootstrapped 95% confidence intervals were calculated using the confint (boot method) function in R. All other calculations were done using R package Lme4.

## Results

The total number of NHs with matched NH residents amounted to 1,617 (72%), see Table 1. The average number of beds per NH was 43.5, and 62.5% of NHs were oriented towards care for NH residents with dementia. Out of 142,600 NH residents with available street addresses, 74,327 were matched to a NH and included in the study. In the group of included NH-residents there were between 48- to 52,000 individuals observed per month and a total of 60,813 ED visits registered during the study period (52 962 monthly observations of one or more ED visits). The NH-residents not included in the study differed from the included residents in terms of age, sex, prevalence of dementia and marital status, see supplementary table S2 and supplementary figure S3.

**Table 1:** Characteristics of included nursing homes

Number of NHs	1617
NH number of beds, mean (SD)	43.54 (22.76)
NHs specialized in care of residents with dementia (%)	1011 (62.5)
NHs with private ownership (%)	303 (18.7)
Registered nurses per NH bed on weekdays, mean (SD)	0.05 (0.03)
Registered nurses per NH bed on weekends, mean (SD)	0.02 (0.03)
Urbanicity (%)	1 = 318 (19.7) 2 = 665 (41.1) 3 = 159 (9.8) 4 = 242 (15.0) 5 = 211 (13.0) 6 = 22 (1.4)

**Fig. 1:**
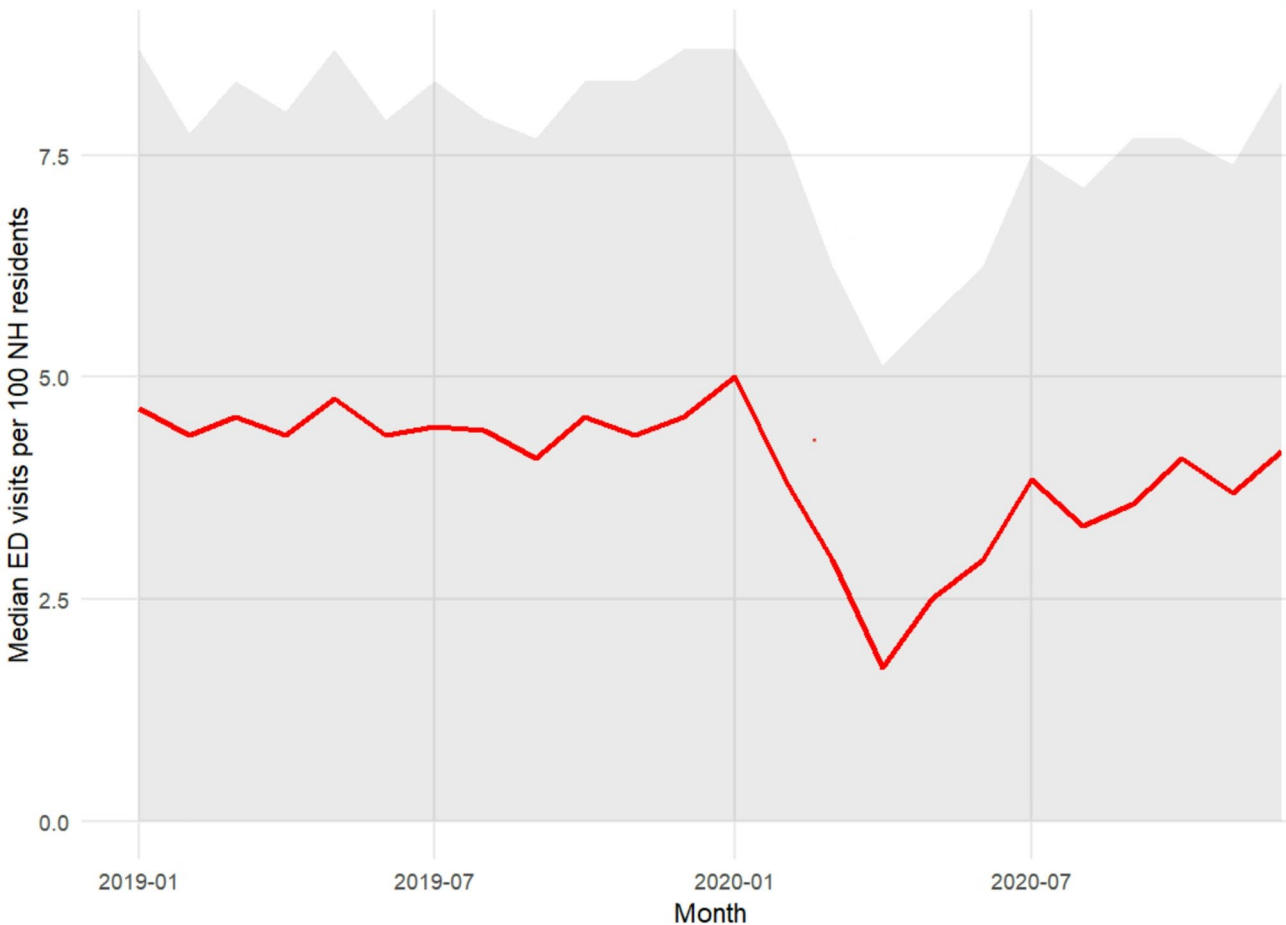
Descriptive graph of median ED visits per 100 NH residents

The median number of ED visits per NH and month ranged between 4.1 (IQR 0–7.6) and 4.8 (IQR 0–8.7) per 100 NH residents in 2019, see Fig. 1. In 2020, the monthly median number of ED visits ranged between 1.7 (IQR 0–5.1) to 5.0 (IQR 0–8.5) due to a drop in ED visits during the first wave of the pandemic, see Fig. 1. Described as yearly incidence rates, the median incidence rate of ED visits from NHs was 5.2 per 100 person-months in 2019 (IQR = 3.7–6.9) and 4.4 per 100 person-months in 2020 (IQR = 3.0–5.7), see supplementary figure S4.

Median number of ED visits per NH and month (standardized to ED visits per 100 NH residents) shown in red. Grey ribbon represents the interquartile range.

Bivariate regression models indicated that NHs with private ownership had higher odds of their residents experiencing ED visits compared to public NHs. NHs specialized in dementia care, NHs in more rural municipalities and NHs with a medium number of nurses had lower odds of ED visits. These findings were consistent in the adjusted models (M1-M3) with the exception of private NHs that did not have higher odds of ED visits after adjusting for the number of nurses and characteristics of NH residents (see Table 2).

The fourth model (M4) included an interaction term for the pandemic and during the first year of the pandemic, a non-significant trend towards a decrease in ED visits was observed (OR = 0.82).

Private nursing homes had higher odds of ED visits before the pandemic (OR = 1.06), which then decreased during the pandemic (OR = 0.94), see Table 2. The effects of the aggregated individual variables are presented in the supplement (Supplementary table S3).

Between group variance for NHs regarding ED visits accounted for 3.3% of the variance in the null model (see Table 2) which decreased to 2.7% in M4 (see Table 3). Random intercept for month was negligible (not reported). Pseudo-R2 values indicated that M1-M4 explained roughly 55.1–55.5% of the variation in the data compared to the null-model (M0).

**Table 2:** Multi-level logistic regression analysis showing odds ratios and confidence intervals of ED visits among NHs during 2019–2020

	BIVARIATE (95% CI)	M1 (95% CI)	M2 (95% CI)	M3 (95% CI)
Intercept		0,04 (0,04 − 0,05)	0,05 (0,04 − 0,05)	0,05 (0,04 − 0,06)
Private ownership (ref = public)	1,06 (1,01–1,11)	1,04 (1,00–1,10)	1,03 (0,98 − 1,09)	1,04 (0,99 − 1,10)
Dementia profile (ref = somatic)	0,91 (0,88 − 0,95)	0,89 (0,85 − 0,92)	0,88 (0,85 − 0,92)	0,90 (0,87 − 0,94)
Medium size (ref = small)	0,98 (0,94-1.03)	0,97 (0,93 − 1,03)	0,97 (0,93 − 1,02)	0,97 (0,93 − 1,02)
Large size	1,00 (0,95 − 1,05)	0,99 (0,94 − 1,04)	0,99 (0,94 − 1,04)	0,98 (0,94 − 1,03)
Urbanicity 2 (ref = most urban)	1,04 (0,99 − 1,11)	1,03 (0,99 − 1,08)	1,03 (0,98 − 1,09)	1,06 (1,00–1,11)
Urbanicity 3	0,96 (0,90 − 1,04)	0,94 (0,87 − 1,01)	0,94 (0,87 − 1,02)	0,97 (0,89 − 1,06)
Urbanicity 4	0,90 (0,85 − 0,97)	0,89 (0,83 − 0,95)	0,89 (0,83 − 0,96)	0,91 (0,85 − 0,98)
Urbanicity 5	0,93 (0,88 − 1,01)	0,9 (0,84 − 0,97)	0,90 (0,83 − 0,96)	0,93 (0,86 − 1,00)
Urbanicity 6	0,51 (0,41 − 0,63)	0,5 (0,42 − 0,61)	0,51 (0,41 − 0,61)	0,54 (0,44 − 0,65)
Medium number of nurses on weekdays (ref = low)	0,97 (0,93 − 1,02)		0,95 (0,91 − 1,00)	0,95 (0,92 − 1,00)
High number of nurses on weekdays	0,99 (0,94 − 1,05)		0,95 (0,90 − 1,01)	0,96 (0,91 − 1,01)
Medium number of nurses on weekends (ref = low)	0,93 (0,90 − 0,98)		0,97 (0,92 − 1,01)	0,97 (0,93 − 1,01)
High number of nurses on weekends	1,01 (0,96 − 1,07)		1,04 (0,99 − 1,10)	1,04 (0,99 − 1,10)
	**AIC**	**NH Variance (PCV)**	**NH VPC**	**Pseudo-R2 total (fixed effects)**
M0	107,080	0,111 (ref)	3,3%	
M1	106,985	0,102 (8,1%)	3,0%	55,5% (4,9%)
M2	106,981	0,101 (9,0%)	3,0%	55,5% (5,3%)
M3	106,931	0,0897 (19,2%)	2,7%	55,1% (7,4%)

*CI = confidence interval, VPC = variance partition coefficient, AIC = Akaike Information Criterion. M0: random intercept of the variation in ED visits between NHs. BIVARIATE: bivariate regression of organizational variables. M1: M0 + organizational variables. M2: M1 + number of nurses. M3: M2 + morbidity and socioeconomic status (see supplementary table S3)

**Table 3:** Multi-level logistic regression analysis showing odds ratios and confidence intervals of ED visits before and after start of COVID-19 pandemic

	M4 (95% CI)	COVID-19 interaction (95% CI)
Intercept	0,06 (0,05 − 0,07)	
Private ownership (ref = public)	1,06 (1,01–1,12)	0,94 (0,89 − 0,98)
Dementia profile (ref = somatic)	0,90 (0,87 − 0,94)	1,00 (0,96 − 1,04)
Medium size (ref = small)	0,98 (0,94 − 1,03)	0,96 (0,91 − 1,01)
Large size	0,99 (0,94 − 1,04)	0,99 (0,94 − 1,04)
Urbanicity 2 (ref = most urban)	1,05 (0,99 − 1,11)	1,02 (0,96 − 1,08)
Urbanicity 3	0,99 (0,90 − 1,07)	0,95 (0,88 − 1,03)
Urbanicity 4	0,92 (0,85 − 0,99)	0,97 (0,90 − 1,05)
Urbanicity 5	0,94 (0,87 − 1,02)	0,95 (0,87 − 1,03)
Urbanicity 6	0,59 (0,47 − 0,72)	0,77 (0,59 − 0,98)
Medium number of nurses on weekdays (ref = low)	0,94 (0,89 − 0,98)	1,04 (1,00–1,09)
High number of nurses on weekdays	0,95 (0,89 − 1,00)	1,02 (0,97 − 1,08)
Medium number of nurses on weekends (ref = low)	0,96 (0,91 − 1,00)	1,02 (0,98 − 1,07)
High number of nurses on weekends	1,04 (0,98 − 1,10)	1,00 (0,95 − 1,05)
Pandemic	0,82 (0,67 − 1,01)	
AIC	106,922	
NH Variance (PCV)	0,090 (18,9%)	
NH VPC	2,7%	
Pseudo-R2 total (fixed effects)	55,3% (12,2%)	

CI = confidence interval, VPC = variance partition coefficient, AIC = Akaike Information Criterion. M4: M3 + Interaction effect of pandemic

## Discussion

This study aimed to characterize the variation in ED visits from NHs in Sweden. Using individual level patient data cross-linked to nursing home addresses, this study is the first to describe the rate of ED visits from individual NHs in Sweden. The annual incidence rates of ED visits from individual NHs demonstrated a variation similar to that observed in previous studies [7, 16, 28]. The odds of ED visits for NH residents living in NHs specialized in dementia care were consistently lower than compared to those in somatic NHs. For this group, the odds of ED visits were 11–12% lower in models 1 and models 2, with a slight increase after adjusting for morbidity and socioeconomic variables (OR = 0.90). These findings align with previous studies, which suggest that patients with dementia are less likely to be referred to EDs [20–22].

The effects of NHs being located in urban versus rural areas suggest that NHs in rural areas have lower odds of their NH residents experiencing ED visits [24, 33]. While this aligns with previous research, the effect is particularly pronounced in NHs located in the most rural municipalities (OR = 0.54) [27]. Further research is needed to investigate whether the most rural NHs have compensated for the longer distances to EDs by increasing their own medical capacity (e.g., reliance on mobile care services) or if there is an unmet medical need among these NH residents.

Consistent with previous research, privately owned NHs appeared to have higher odds ratios for ED visits in comparison to public NHs in the bivariate analysis [17, 32, 34, 35]. However, this effect was no longer statistically significant after adjusting for number of nurses in model 3, suggesting that the effect of private ownership is mediated through staff density. Further research is needed to determine whether there are differences in potentially avoidable ED visits between private and public NHs.

The effect of the number of nurses on ED visits was less substantial in this study compared to previous studies [30–32, 34]. The ORs indicate that NHs with a medium number of nurses may have experienced fewer ED visits, but the results were not statistically significant. Furthermore, the NHs with the highest number of nurses on weekends showed increased odds of ED visits (not statistically significant), suggesting a non-linear relationship between staff density and ED visits. This may be due to increased detection of medical emergencies requiring ED services on weekends [30–32, 34]. Contrary to previous studies, NH size did not have any impact on ED visits, despite previous studies showing both higher rates of infections (e.g. COVID-19) and ED visits for larger NHs [24, 29].

Finally, the VPC showed that between 2.5-3.3% of the variation in ED visits are attributable to differences between NHs. The impact of NH characteristics on the total variation in ED visits is therefore rather limited. However, as the population of NH residents is large, these differences may still affect thousands of individuals over time.

### Impact of the COVID-19 pandemic

As the study period includes the initial period of the COVID-19 pandemic, the secondary research objective was to explore the effects of COVID-19 on ED-visits. As seen in Fig. 1, incidence rates of ED visits remained relatively constant before the pandemic and then decreased substantially during the first pandemic wave. However, the decrease in ED visits during the initial phase of the pandemic was not statistically significant, likely indicating large variation between different Swedish regions.

Furthermore, as the COVID-19 pandemic was an external chock to the health care system, the results may be biased from changes in health service provision in NHs during the pandemic. However, the interaction effects in the model adjusting for organizational characteristics, NH-resident characteristics and the pandemic (M4) suggests that the effects of NH-characteristics in the pre-pandemic period were similar to the pandemic period, indicating that the pandemic did not have differential effects on ED visits in relation to NH-characteristics.

However, there were some exceptions. Privately owned NHs had statistically significant increased odds of ED visits (OR = 1.06) in the pre-pandemic period, although this effect decreased during the pandemic (OR = 0.94). This finding aligns with previous research, which has shown that private NHs tend to be more responsive to guidelines compared to public NHs, in this case, following recommendations from regional health care systems to avoid transferring clinically frail patients to ERs [48]. Finally, the odds ratio of ED visits in NHs located in rural municipalities decreased even further during the pandemic (OR = 0.77). This might be attributed to these areas having a lower population density and subsequently lower rates of COVID-19 infections that needed treatment in EDs. However, as the variable urbanicity approximates both distance to hospital and spread of COVID-19, it is not possible to disentangle the impact of COVID-19 from potential effects of the distance to hospital during the pandemic.

### Limitations

This study has several data limitations that are important to consider when interpreting the results. First, only 55% of NH residents with addresses registered in December 2018, 2019 or 2020 could be matched to addresses of specific NHs. As described in supplementary table S2, non-matched NH residents differed from the included NHs in several important aspects (such as age and dementia). The generalizability of the results from this study is therefore uncertain as the population of included NH-residents may not represent an accurate measure of the average ED visits from the studied NHs.

Second, the self-reported data from NHs was only available for 2019 and it is not known to what extent indicators such as number of beds and number of nurses changed during 2020. Furthermore, the self-reported data does not include all variables that could be important in order to explain variations in ED visits (such as the presence of short-term beds). Although the self-reported data has been used in other scientific studies, the validity of this data is not certain [48].

Finally, the lack of data regarding primary care services provided by physicians is a concern as these services are likely to have an impact on ED-visit rates. This, together with other unmeasured confounding, are probably impacting the model’s predictive accuracy, see supplementary figure S1. In order to improve predictive accuracy, future studies should include indicators of primary care availability and continuity of care.

## Conclusion

Organizational characteristics such as ownership and geographic location of Swedish NHs may influence ED-visit rates. Some of the results align with previous studies as NHs specialized in dementia care and located in rural municipalities had lower odds of ED visits for their residents. However, in contrast to previous studies this study did not find any consistent impact of NH size, ownership or nurse density on ED visits.

The findings of this study may hold important implications for the future development of both health and social care for NH residents. By identifying the potential causes of variation in ED-vist rates, policymakers and health care providers may be able to address the underlying factors contributing to these variations. This could lead to improvements in the quality of care within NHs, ensuring that residents receive more timely and appropriate care, potentially reducing unnecessary ED visits. Such improvements could also have broader impacts on health equity by ensuring that all NH residents, regardless of their background or the type of facility they reside in, have equal access to high quality care. Additionally, understanding these variations could potentially enhance pandemic preparedness by providing insights into how nursing home care and health systems can adapt to ensure continued access to essential services during future pandemics.

## Supplementary Information

Below is the link to the electronic supplementary material.

Supplementary Material 1

## Data Availability

**Nursing homes** Data regarding NHs are publicly available at the National Board of Health and Welfares website (In Swedish, https://www.socialstyrelsen.se/statistik-och-data/oppna-jamforelser/socialtjanst/aldreomsorg/hemtjanst-och-sarskilt-boende). Nursing home addresses to the NHs included in the above surveys may be requested from NBHW. Municipal classifications of urbanicity is publicly available at the Swedish Agency for Economic and Regional Growth, see https://tillvaxtverket.se/tillvaxtverket/statistikochanalys/statistikomregionalutveckling/regionalaindelningar/staderochlandsbygder.1844.htm. **Nursing home residents** The data regarding individual NH residents is considered sensitive data and is not publicly available. Furthermore, data cannot be made available as the General Data Protection Regulation, The Swedish law SFS 2018:218, The Swedish Data Protection Act, the Swedish Ethical Review Act and the Public Access to Information and Secrecy Act asserts that these types of sensitive data can only be made available to researcher who meet the criteria for access after legal review by the Ethical Review Authority of Sweden. Please contact the first author for additional details and the possibility of making aggregated data available.

## Abbreviations

ED: Emergency Department
NH: Nursing home
