# Supplementary material for: Variation in emergency department visits among residents of Swedish nursing homes between 2019 and 2020, a population-based cohort study
Source: BMC Health Serv Res. 2025 Sep 10;25:1196. doi: 10.1186/s12913-025-13443-9 (PMC12421770; doi:10.1186/s12913-025-13443-9)
Supplement: Supplementary file 1 — Supplementary Material 1 [file 12913_2025_13443_MOESM1_ESM.docx]

# Supplementary material

## Goodness of fit


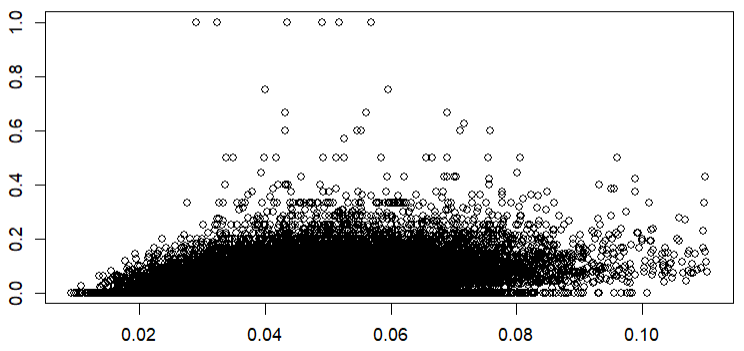


**Figure S1. Plot of observed (Y-axis) versus predicted (X-axis) values based on the multivariate regression in M1.**


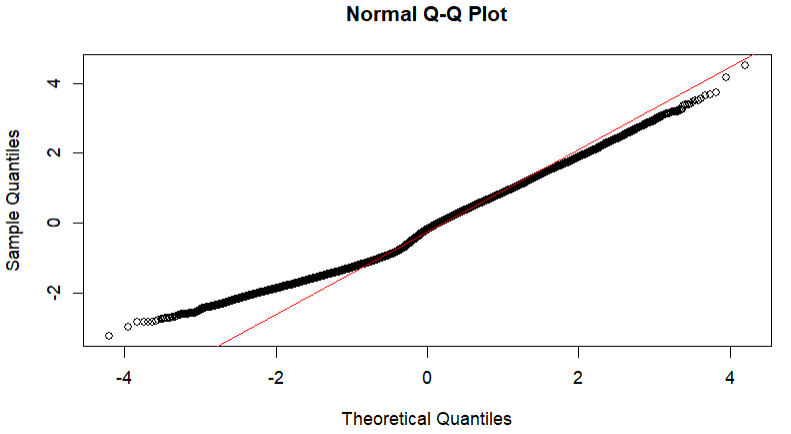


**Figure S2. Q-Q plot of residuals based on the multivariate regression in M1**

**Supplementary table S1. Data sources**

| **Data** | **Registry (source)** | **Variable** |
| --- | --- | --- |
| Living in NH | Swedish Social Service Register (NBHW) | Boform |
| Age | RTB (SCB) |  |
| Sex | RTB (SCB) |  |
| ED-visit | National Patient Registry. Specialized outpatient care (NBHW) | VERKS_AKUT |
| Main diagnosis at ED-visit | National Patient Registry. Specialized outpatient care (NBHW) | HDIA |
| Morbidity | National Patient Registry. Inpatient and specialized outpatient care (NBHW)  Swedish Drug Prescription Registry (NBHW) | HDIA, DIAGNOS  ATC (dementia = N06D) |
| Education | LISA (SCB) |  |
| Civil status | LISA (SCB) |  |
| Country of Birth | LISA (SCB) |  |
| Addresses of NH-residents | SCB | Folkbokföringsadress |
| Addresses of NHs | Business register (SCB)  Unit survey (NBHW) |  |
| NH characteristics data | Unit Survey (NBHW) |  |

## Example of model specification (M4)

glmer(cbind(ED visit, no ED visit) ~ (private + profile + size + urbanity + nurse weekday + nurse weekend + mean ED risk score + proportion born in Sweden + proportion married + proportion highest education) * pandemic + (1| nursing home) + (1|month), family = binomial, control = glmerControl(optimizer = “bobyqa”, optCtrl = list(maxfun = 1e5)

## Description of dataset

**Supplementary Table S2. Characteristics of NH-residents.**

|  | **Included (matched to NH)** | **Excluded (Not matched to NH)** |
| --- | --- | --- |
| N (% of total) | 74, 327 (%) | 68,273 (%) |
| Mean age (SD) | 86.12 (7.76) | 84.73 (7.97) |
| Female (%) | 51,531 (69.3) | 40,343 (59.1) |
| Dementia (%) | 30,045 (40.4) | 29,103 (42.6) |
| Share of NH-residents with at least one ED-visit (%) | 32,229 (43.4) | 29,729 (43.5) |
| Mortality (%) | 29,106 (39.2) | 32,010 (46.9) |
| SES – civil status | Married = 8,537 (11.5)  Divorced = 14,755 (19.9)  Unmarried = 7,940 (10.7)  Widow/widower = 43,095 (58.0) | Married = 19,044 (28.5)  Divorced = 11,102 (16.6)  Unmarried = 7141 (10.7)  Widow/widower = 29,648 (44.3) |
| SES Born in Sweden | Sweden = 65,737 (88.4)  Outside of Sweden =  8587 (11.6) | Sweden = 60,264 (88.3)  Outside of Sweden = 8008 (11.7) |
| SES highest education | Primary = 38,288 (51.5)  Secondary = 25,799 (34.7)  Tertiary = 10,197 (13.7) | Primary = 31,262 (46.7)  Secondary = 23,925 (35.8)  Tertiary = 11,703 (17.5) |
| **Most common disease chapter of primary diagnosis at ED visit (% of total)** |  |  |
| *Symptoms, signs and abnormal clinical and laboratory findings not elsewhere classified* | 14,935 (27.0) | 15,757 (27.3%) |
| *Injury, poisoning and certain other consequences of external causes* | 13,039 (23.6) | 12,051 (20.8) |
| *Diseases of the circulatory system* | 3642 (6.6) | 4068 (7.0) |
| *Diseases of the respiratory system* | 2801 (5.1) | 2945 (5.1) |
| *Diseases of the musculoskeletal system and connective tissue* | 2751 (5.0) | 2769 (4.8) |
| *Certain infectious and parasitic diseases* | 2728 (4.9) | 2803 (4.8) |
| *Factors influencing health status and contact with health services* | 2505 (4.5) | 2929 (5.1) |
| *Diseases of the genitourinary system* | 2221 (4.0) | 2460 (4.3) |
| *Covid-19* | 502 (0.9) | 530 (0.9) |
|  |  |  |

**
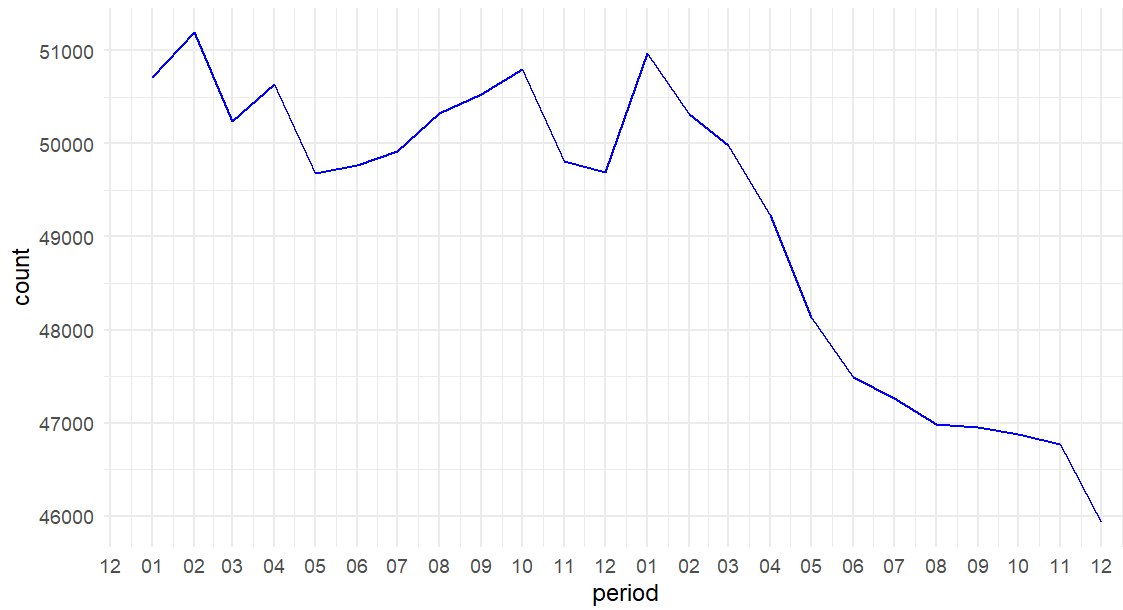
**

**Supplementary figure S3. Number of NH-resident observations per month**

**
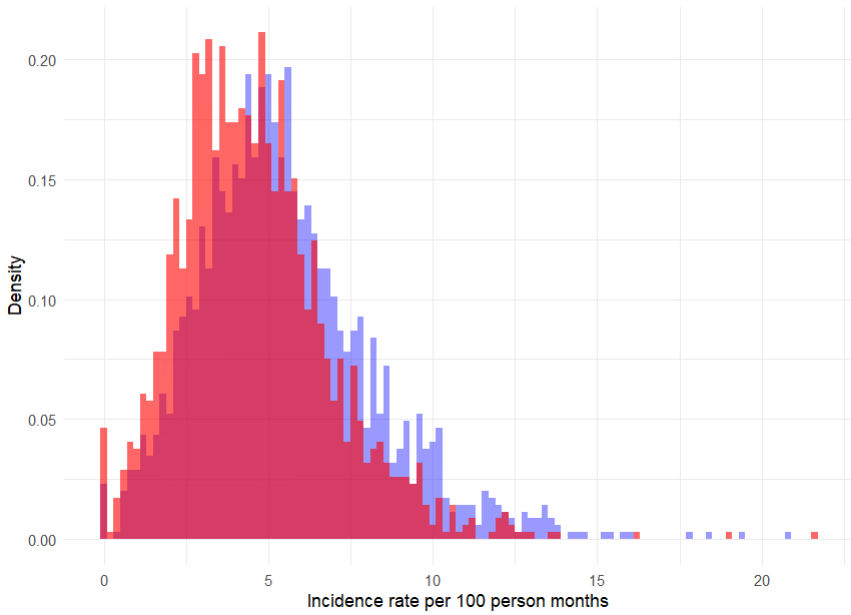
**

**Supplementary Figure S4. Overlapping density ploy of ED incidence rate from nursing homes in 2019 (blue) and 2020 (red) per 100 person months.**

**Supplementary table S3. M1-M3 including the effects of the socioeconomic variables and the ED risk prediction score used in M3 and M4.**

|  | BIVARIATE (95% CI) | M1 (95% CI) | M2 (95% CI) | M3 (95% CI) |
| --- | --- | --- | --- | --- |
| Intercept |  | 0,04 (0,04-0,05) | 0,05 (0,04-0,05) | 0,05 (0,04-0,06) |
| Private ownership (ref = public) | 1,06 (1,01-1,11) | 1,04 (1,00-1,10) | 1,03 (0,98-1,09) | 1,04 (0,99-1,10) |
| Dementia profile (ref = somatic) | 0,91 (0,88-0,95) | 0,89 (0,85-0,92) | 0,88 (0,85-0,92) | 0,9 (0,87-0,94) |
| Medium size (ref = small) | 0,98 (0,94-1,03) | 0,97 (0,93-1,03) | 0,97 (0,93-1,02) | 0,97 (0,93-1,02) |
| Large size | 1,00 (0,95-1,05) | 0,99 (0,94-1,04) | 0,99 (0,94-1,04) | 0,98 (0,94-1,03) |
| Urbanity 2 (ref = most urban) | 1,04 (0,99-1,11) | 1,03 (0,99-1,08) | 1,03 (0,98-1,09) | 1,06 (1,00-1,11) |
| Urbanity 3 | 0,96 (0,90-1,04) | 0,94 (0,87-1,01) | 0,94 (0,87-1,02) | 0,97 (0,89-1,06) |
| Urbanity 4 | 0,90 (0,85-0,97) | 0,89 (0,83-0,95) | 0,89 (0,83-0,96) | 0,91 (0,85-0,98) |
| Urbanity 5 | 0,93 (0,88-1,01) | 0,9 (0,84-0,97) | 0,9 (0,83-0,96) | 0,93 (0,86-1,00) |
| Urbanity 6 | 0,51 (0,41-0,63) | 0,5 (0,42-0,61) | 0,51 (0,41-0,61) | 0,54 (0,44-0,65) |
| Medium number of nurses on weekdays (ref = low) | 0,97 (0,93-1,02) |  | 0,95 (0,91-1,00) | 0,95 (0,92-1,00) |
| High number of nurses on weekdays | 0,99 (0,94-1,05) |  | 0,95 (0,90-1,01) | 0,96 (0,91-1,01) |
| Medium number of nurses on weekends (ref = low) | 0,93 (0,90-0,98) |  | 0,97 (0,92-1,01) | 0,97 (0,93-1,01) |
| High number of nurses on weekends | 1,01 (0,96–1,07) |  | 1,04 (0,99-1,10) | 1,04 (0,99-1,10) |
| ED prediction score |  |  |  | 1,06 (1,04-1,08) |
| Born in Sweden (proportion) |  |  |  | 0,84 (0,71-0,98) |
| Married (proportion) |  |  |  | 1,03 (0,84-1,26) |
| Tertiary education (proportion) |  |  |  | 0,81 (0,66-0,99) |
|  |  |  |  |  |
|  |  |  |  |  |
|  |  |  |  |  |
|  | AIC | NH Variance | NH VPC | Pseudo-R2 total (fixed effects) |
| M0 | 107080 | 0,111 | 3,3% |  |
| M1 | 106985 | 0,102 | 3,0% | 55,5% (4,9%) |
| M2 | 106981 | 0,101 | 3,0% | 55,5% (5,3%) |
| M3 | 106931 | 0,0897 | 2,7% | 55,1% (7,4%) |
